# Loss of Interleukin-6 Influences Transcriptional Immune Signatures and Alters Bacterial Colonization in the Skin

**DOI:** 10.3389/fmicb.2021.658980

**Published:** 2021-07-06

**Authors:** Lerin R. Luckett-Chastain, Catherine J. King, William M. McShan, Jenny R. Gipson, Allison F. Gillaspy, Randle M. Gallucci

**Affiliations:** ^1^Department of Pharmaceutical Science, University of Oklahoma Health Science Center, Oklahoma City, OK, United States; ^2^College of Medicine Core Facilities, University of Oklahoma Health Science Center, Oklahoma City, OK, United States; ^3^Department of Microbiology and Immunology, University of Oklahoma Health Science Center, Oklahoma City, OK, United States

**Keywords:** RNA sequencing, microbiome, T helper 1, T helper 2, interluekin-6, antimicrobial peptides

## Abstract

The skin functions as a protective barrier to inhibit the entry of foreign pathogens, all the while hosting a diverse milieu of microorganisms. Over time, skin cells, immune cells, cytokines, and microbes interact to integrate the processes of maintaining the skin’s physical and immune barrier. In the present study, the basal expression of two immunologically divergent mouse strains C57BL/6 and BALB/c, as well as a strain on the C57 background lacking IL-6, was characterized. Additionally, cutaneous antimicrobial gene expression profiles and skin bacterial microbiome were assessed between strains. Total RNA sequencing was performed on untreated C57BL/6 (control), BALB/c, and IL-6-deficient skin samples and found over 3,400 genes differentially modulated between strains. It was found that each strain modulated its own transcriptional “profile” associated with skin homeostasis and also influenced the overall bacterial colonization as indicated by the differential phyla present on each strain. Together, these data not only provide a comprehensive view of the transcriptional changes in homeostatic skin of different mouse strains but also highlight the possible influence of the strain differences (e.g., Th1/Th2 balance) as well as a role for IL-6 in overall skin immunity and resident microbial populations.

## Introduction

The skin is the most exposed interface with the environment, therefore acting as the body’s first line of physical and immunological defense. Additionally, it comes into contact with a host of pathogenic organisms and provides a home to a myriad of commensal organisms (Belkaid and Segre, 2014). This leads to the formidable tasks of not only warding off foreign pathogens but also maintaining a peaceful symbiotic relationship with resident microbiota. However, this physical barrier is susceptible to injuries and/or damage (*via* irritants, UV radiation, or pathogens) that could allow the entry of opportunistic microbial agents. Fortunately, the innate immune response has evolved to include an arsenal of host defense mechanisms including the (1) production and release of antimicrobial peptides (AMPs) and other proteins with antimicrobial activity, (2) induction of inflammatory cytokines and chemokines, and lastly (3) initiation and modulation of the adaptive immune response.

Epidermal keratinocytes are able to sense and discriminate among microbes that colonize the skin through pattern recognition receptors (PRRs) like toll-like receptors (TLR), mannose receptors, and the NOD-like receptors (Miller, 2008). Additionally, keratinocytes of the deeper epidermal layers synthesize and store AMPs along with various hydrolytic enzymes and polar lipids inside secretory vesicles called lamellar bodies (Braff et al., 2005), and once activation of the PRRs occurs, AMPs can be secreted. Antimicrobial peptides such as S100 family proteins, peptidoglycan-recognition proteins (PGLYRPs), calcium-dependent lectins, elastase inhibitors, structural and membrane receptors, iron metabolism proteins, and various chemokines (Kolls et al., 2008) are amphipathic in nature, enabling them to interact directly with microbes, disrupt lipid membranes, and eventually lead to cell death. Some AMPs are constitutively expressed and detected in both healthy and/or injured epithelium. However, over the past few decades, numerous studies have indisputably shown that various AMPs are associated with chronic inflammatory skin disease conditions such as psoriasis (Morizane and Gallo, 2012), atopic dermatitis (Ong et al., 2002), non-healing wounds, and injury (Harder et al., 2010).

The adaptive immune response can be characterized in various manners such as by cell type or antigen specificity. One useful classification is through describing T helper (Th) cell responses characterize by distinct cytokine production profile. Of the T helper cell responses, it has been shown that the balance between Th1 and Th2 responses are critical in maintaining homeostatic versus diseased states and in disease initiation/progression (Infante-Duarte and Kamradt, 1999). Variation of this Th1/Th2 balance is reported to be demonstrated by the apparent difference in immune responses between the C57BL/6 and BALB/c mouse strains. T cells from C57BL/6 mice preferentially produce Th1 cytokines (IFN-γ), while those from BALB/c favor high Th2 cytokine production (IL-4) (Watanabe et al., 2004). Interestingly, *in vitro* studies have shown that macrophages from these mouse strains exert differing responses to lipopolysaccharide (Mills et al., 2000) and overall have different innate immune responses during bacterial infections (Watanabe et al., 2004).

Interleukin-6 (IL-6) has been variously classified as Th2 or pro-inflammatory, but what is certain is that it is extremely pleiotropic (Klimpel, 1980; Yasukawa et al., 1987). This cytokine has hormone-like attributes that affect numerous aspects of vascular disease, lipid metabolism, insulin resistance, mitochondrial activities, the neuroendocrine system, and neuropsychological behavior (McInnes and Schett, 2007; Jones et al., 2011; Rohleder et al., 2012; Hunter and Jones, 2017). Interleukin-6 is produced by many different cell types (both in immune and non-immune associated cells), and while it is well known to have a protective role in many infections, these same activities may be the key to the switch from acute to chronic inflammation. Diseases and malignancies associated with elevated IL-6 levels are numerous including CAD and abdominal aortic aneurysms, as well as autoimmune disorders such as systemic lupus erythematosus and rheumatoid arthritis (Woo and Humphries, 2013). Furthermore, IL-6 is closely linked to skin wound healing (Gallucci et al., 2000; Lin et al., 2003) and appears to modulate the stratum corneum regeneration and skin barrier function associated with maintaining skin homeostasis (Wang et al., 2004). In addition, IL-6 modulates the systemic immune defense mechanism against the bacterial pathogens *Listeria monocytogenes* (Dalrymple et al., 1995) and *Mycobacterium tuberculosis* (Ladel et al., 1997) and, along with IL-1, enhances the production of AMPs in composite keratinocyte grafts (Erdag and Morgan, 2002). Additionally, IL-6 has been shown to have a crucial role in the inflammatory response against Gram-negative bacteria (Nibali et al., 2008) and shown to be present in endothelial cells, fibroblasts, and macrophages of subjects with periodontitis as compared with healthy individuals (Takahashi et al., 1994). Conversely, IL-6 inhibits IL-1 and TNF-α in monocytes/macrophages (Aderka et al., 1989) and protects against tissue inflammation in some models (Ulich et al., 1991; Lee et al., 2013) further indicating an anti-inflammatory role.

It has been previously recognized that genotype is an important determinant of host susceptibility to major human diseases, including infections (Gruenheid and Gros, 2010; Chapman and Hill, 2012). In the present study, our goal was to investigate the potential influence of genetic predisposition and the role of IL-6 on the transcriptional and microbial changes in homeostatic skin. Therefore, deep sequencing was performed on normal, homeostatic skin isolated from three different mouse models (BALB/c, C57BL/6, and IL-6 deficient on the C57 background) to generate basal transcriptional profiles as well as determine an associated “normal” microbiome. These models in essence compare two strains that differ on the polygenic level, C57 Th1-predisposed (control) versus a polygenic Th2-predisposed (BALB/c) model, and then furthermore, this control strain is contrasted to itself albeit missing a single highly pleiotropic cytokine gene (IL-6 deficient). It was shown that not only are their different immune profiles associated with each strain, but the classes of AMPs transcribed varied, as well as the populations of bacteria that colonize the skin differed quite dramatically despite apparently healthy skin morphology. These data not only provide a novel database to use for the further understanding of skin maintenance and commensal microbiome under diverse immune predispositions but also highlight the pleiotropic and central role of IL-6.

## Materials and Methods

### Mice

IL-6KO (B6.129S2-*Il6^TM 1*Kopf*^*/J on C57BL/6J background), BALB/c, and C57BL/6J (WT) mice, 8–12 weeks old, were acquired from the Jackson Laboratory (Bar Harbor, ME, United States). The IL-6KO mice were created from a C57BL/6 background. Mice were group-housed in polycarbonate cages containing hardwood chip bedding at room temperature (21 ± 3°C) on a 12-h light/dark cycle. Animals were allowed to acclimate to the AAALAC-accredited animal facility for at least 1 week before the experiment began. Mice were then sedated with isoflurane and ∼9 cm^2^ section of fur was clipped. Seven days after hair removal, skin samples were collected *via* a 4-mm full thickness punch biopsy. Each experimental group contained six mice and were collected simultaneously (C57, BALB/c, and IL-6KO). Throughout the studies, animals received humane care according to the criteria outlined in the *Guide for the Care and Use of Laboratory Animals* prepared by the National Academy of Sciences and published by the National Institutes of Health.

### Illumina RNA-Seq Libraries

RNA-seq libraries were constructed using the Illumina TruSeq RNA LT v2 kit and established protocols. The library construction was done using total RNA isolated from mice skin tissue (1 μg). RNA quality for each prep was analyzed prior to library construction using the Agilent Bioanalyzer 2100 and Agilent RNA Nano total RNA chips. Each library was indexed during library construction in order to multiplex for sequencing on the Illumina MiSeq platform. Samples were sequenced in batches of three libraries per 2 × 150 bp paired end sequencing run on the Illumina MiSeq. On average, a total of 40 million reads (6 Gb) of sequencing data were collected per run. Raw data for each sample were analyzed using CLC Genomics Workbench software from Qiagen (Redwood City, CA, United States, formerly CLC Bio). Raw sequence reads were mapped to the *Mus musculus* genome for the identification of genes expressed under each condition. Pairwise comparison of the expression results was performed using the total mapping results for BALB/c versus C57BL/6 and IL-6KO versus C57BL/6. Differential gene lists were created using the “Differential Expression for RNA-Seq” tool in the CLC Genomics Workbench software which performs a statistical differential expression test based on a negative binomial generalized linear model (GLM). A two-fold expression cutoff (and FDR *p*-values of <0.1) was used to identify genes that were up- or downregulated under each condition. This stringent cutoff was used to allow for a more definitive and concise list of genes that were modulated in each comparison after *t*-test statistical measures were performed. Each experimental group (C57, BALB/c, and IL-6KO) had an *n* = 6. A gene was considered differentially expressed if the false discovery rate (FDR) for differential expression was less than 0.1 and the fold change was at least two-fold (log_2_ fold change (LFC) of >1). GO enrichment analysis was performed by using the publicly available DAVID Resource^1^ (Mi et al., 2017) and PANTHER databases^2^.

### 16S rRNA (or Microbiome) Sequencing

Skin biopsies were collected as previously stated and flash frozen. Bacterial genomic DNA was extracted from mouse skin using the Quick-DNA Universal Kit and Universal Quick-DNA BashingBead Module (2.0) (Zymo Research, Irvine, CA, United States) using the manufacturer’s protocol with the following modifications. Approximately 50 mg of thawed, homogenized skin was ground in 250 μl of lysis solution using a VWR Pellet Mixer (VWR, Radnor, PA, United States) and a 1.5-ml pestle (Argos Technologies, Elgin, IL, United States) for two repetitions of 30 s each. The remaining volume of lysis solution (500 μl) was added to the tube and vortexed, and the supernatant was transferred to an Eppendorf Safe-Lock microcentrifuge tube. The beads from the ZR BashingBead Lysis tubes were transferred to the tube with the supernatant, and the contents were homogenized using a Bullet Blender Blue (Next Advance, Averill Park, NY, United States) at a speed of 10 for 5 min at 4°C. The tubes were then centrifuged for at 12,000 × *g* for 1 min and DNA from the supernatant was extracted and cleaned as per the Quick-DNA Universal Kit directions.

A 1,465-bp region of 16S rRNA bacterial DNA was amplified from 400 ng of extracted DNA using 25 μl reactions consisting of 0.4 μM each of the primers 27F (5′-AGAGTTTGATCCTGGCTCAG-3′) and 1492R (5′-TACCTTGTTACGACTT-3′) (manufactured by IDT, San Jose, CA, United States); 0.05 U/μl GoTaq Flexi DNA Polymerase, 1 × Colorless GoTaq Flexi Buffer, and 1.5 mM magnesium chloride solution (Promega, Madison, WI, United States); 0.2 mM dNTP (Omega Bio-Tek Inc., Norcross, GA, United States); and 0.1 μg/μl BSA (New England BioLabs, Ipswich, MA, United States). PCR was carried out in T100 (Bio-Rad, Hercules, CA, United States) or TC-312 (Techne, Inc., Burlington, NJ, United States) thermocyclers using an initial denaturation at 95°C for 3 min; 25 cycles each consisting of denaturation at 95°C for 1 min, annealing at 45°C for 1 min, and elongation at 72°C for 1 min; and a final elongation step at 72°C for 5 min. This initial PCR was performed to confirm bacterial DNA extraction and to rule out any erroneous non-bacterial amplicons (Frank et al., 2008). A nested PCR was done using primers targeting the V3–V4 region of the 16S rRNA gene and containing an adapter overhang nucleotide sequence as recommended by Illumina. The 550-bp PCR product was amplified using 0.2 μM each of the primers 16S-F (5′-TCGTCGGCAGCGTCAGATGTGTATAAGAGACAGCCTACG GGNGGCWGCAG-3′) and 16S-R (5′-GTCTCGTGGGCT CGGAGATGTGTATAAGAGACAGGACTACHVGGGTATCTA ATCC-3′) (IDT); 0.05 U/μl GoTaq Flexi DNA Polymerase, 1 × Colorless GoTaq Flexi Buffer, and 1.5 mM magnesium chloride solution (Promega); and 0.2 mM dNTP (Omega Bio-Tek Inc.). PCR was carried out in T100 (Bio-Rad) or TC-312 (Techne, Inc.) thermocyclers using an initial denaturation at 95°C for 5 min; 25 cycles each consisting of denaturation at 95°C for 30 s, annealing at 55°C for 30 s, and elongation at 72°C for 30 s; and a final elongation step at 72°C for 5 min. PCR products were cleaned using the SV gel and PCR cleanup kit (Promega). Negative or “blank” samples were also accessed for each strain to ensure that no potential contamination of reagents occurred during the extraction or amplification process and in cases that no PCR products were amplified (data not shown). Cleaned V3–V4 DNA was sent to the OUHSC Laboratory for Molecular Biology and Cytometry Research (LMBCR) for multiplex, 2 × 300 bp paired end sequencing on the Illumina MiSeq using standard protocols. Libraries were generated using the standard Illumina 16S sequencing workflow and results were analyzed using the CLC Microbial Genomics Module. Briefly, raw sequence reads were analyzed using the CLC Microbial Genomics Module Amplicon-based analysis workflow. First adapter trimming and operational taxonomic unit (OTU) clustering were performed with total reads from each sample. Unique OTUs were compared with the GreenGenes 16S rRNA gene database and taxonomic identifications were made. Differential abundance of the OTUs across different groups was assessed using linear discriminant effect size (LEfSe) version 1.0 (Segata et al., 2011). The data were reported at the level of overall phylum abundance and LDA scores and in the form of a cladogram for each experimental group. Additional OTU information is found *via* accession number PRJNA692120 on the SRA/BioProject database.

## Results

### Overall Gene Expression Profiles and Functional Overview in Normal Skin of BALB/c, C57BL/6, and IL-6KO Mice

**FIGURE 1:**
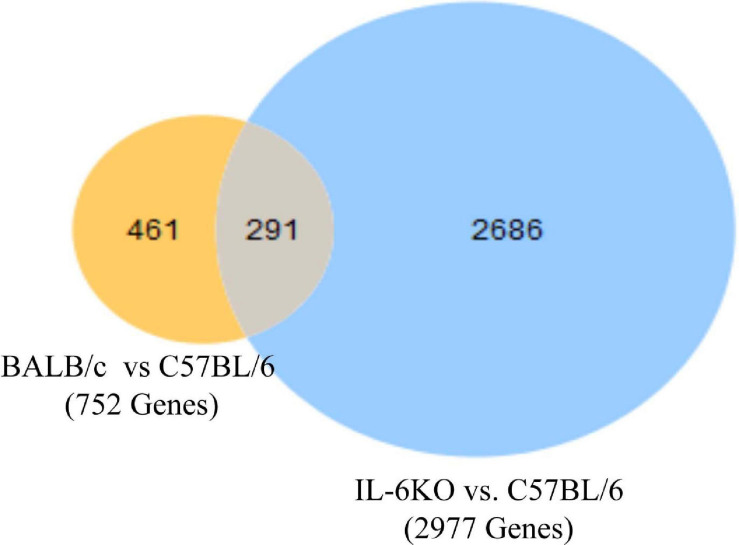
Overall cutaneous gene expression changes in BALB/c, C57, and IL-6-deficient mice. Total RNA was isolated from untreated C57, BALB/c, and IL-6KO (KO) normal skin samples and subjected to transcriptome analysis by MiSeq. Differential gene lists were created with two-fold expression cutoff and false discovery rate (FDR) < 0.1 (*n* = 6/experimental group). Venn diagrams depicting the number of significantly modulated (FC > 2) transcripts in BALB/c versus C57BL/6 (yellow) and IL-6-deficient versus C57BL/6 control (blue). Transcripts that were common between the two comparisons are shown by the overlap.

**FIGURE 2:**
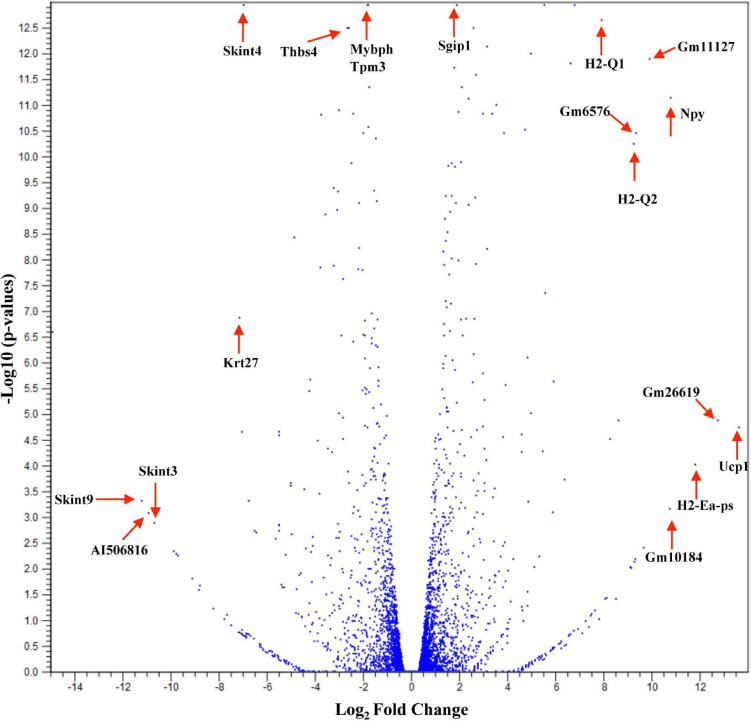
Overall cutaneous gene expression changes in BALB/c versus C57BL/6 skin. Total RNA was isolated from untreated C57 and BALB/c normal skin samples and subjected to transcriptome analysis by MiSeq (*n* = 6/experimental group). Volcano plot comparing gene expression levels in terms of log_2_ fold change versus significance in both group comparisons. The *y*-axis corresponds to the mean expression value of log 10 (*p*-value), and the *x*-axis displays the log_2_ fold change value.

**FIGURE 3:**
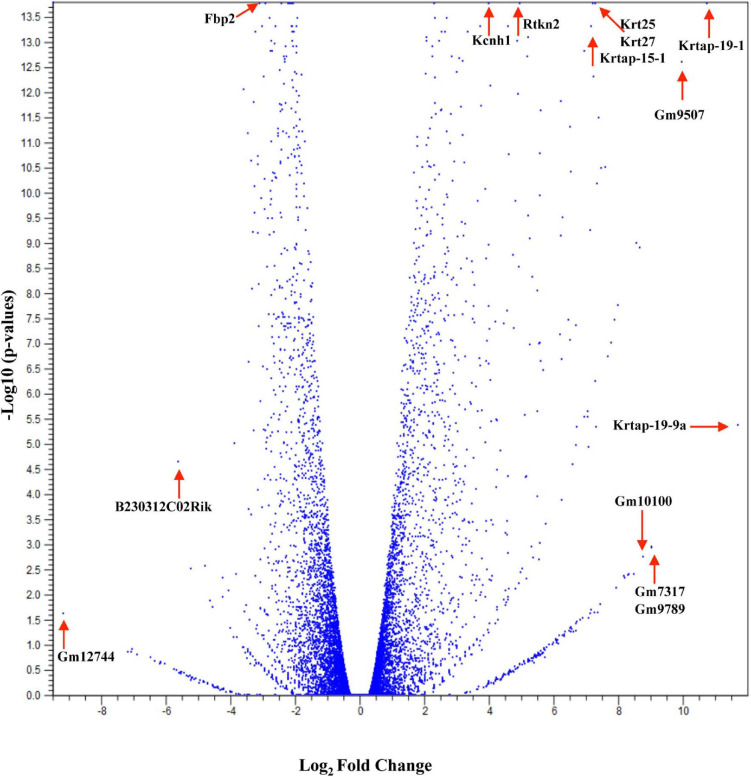
Overall cutaneous gene expression changes in IL-6-deficient versus C57BL/6 control skin. Total RNA was isolated from untreated C57 and IL-6KO (KO) normal skin samples and subjected to transcriptome analysis by MiSeq (*n* = 6/experimental group). Volcano plot comparing gene expression levels in terms of log_2_ fold change versus significance in both group comparisons. The *y*-axis corresponds to the mean expression value of log 10 (*p*-value), and the *x*-axis displays the log_2_ fold change value.

Studies have shown that C57BL/6 (C57) and BALB/c (BALB) mice display prototypical Th1- and Th2-type responses (Watanabe et al., 2004), respectively. While most studies to date have characterized only minor aspects of the immunological differences between these two strains, the goal of these studies was to formulate a more complete and thorough understanding of the overall transcriptional variation in skin associated with each phenotype. Additionally, the absence of IL-6 was investigated to determine what role this single cytokine plays in the immune-associated transcriptional response. Total RNA sequencing was performed on untreated C57, BALB, and IL-6-deficient (KO) skin samples, and it was found that over 3,400 genes were differentially modulated between the three strains (accession number GSE119202) with the majority differentially expressed in IL-6KO as compared with the control strain C57 (Figure 1). To better understand the biological and functional significance, each gene was manually categorized based upon gene ontology term descriptors, published data, and various functional annotation tools (DAVID, PANTHER, and CLC), and it was found that most genes within each comparison (BALB vs. C57 and KO vs. C57) were associated with cellular or metabolic processes although the specific processes differed (Supplementary Figure 1A vs. Supplementary Figure 1B). When gene expression in BALB was compared with C57, over 700 genes were differentially modulated by at least −3- to +3-fold (log scale) as compared with the control with the greatest fold change differences indicated (e.g., *Ucp1*, *H2-Ea-ps*, *H2-Q2*, *Npv*, *Skint3*, *Skint9*) (Figure 2, red arrows). Interestingly, the deletion of IL-6 from the C57 background resulted in nearly four times as many genes that were significantly modulated as compared with BALB and a unique gene expression profile (e.g., *Fbp2*, *Kcnb1*, *Rtkn2*, *Krt25*, *Krt27*, *Krtap19-1*) when compared with its wild type (Figure 3).

### BALB/c, C57, and IL-6KO Mice Display Altered Transcriptional Changes of Immune Response-Associated Genes in Normal Skin

**FIGURE 4:**
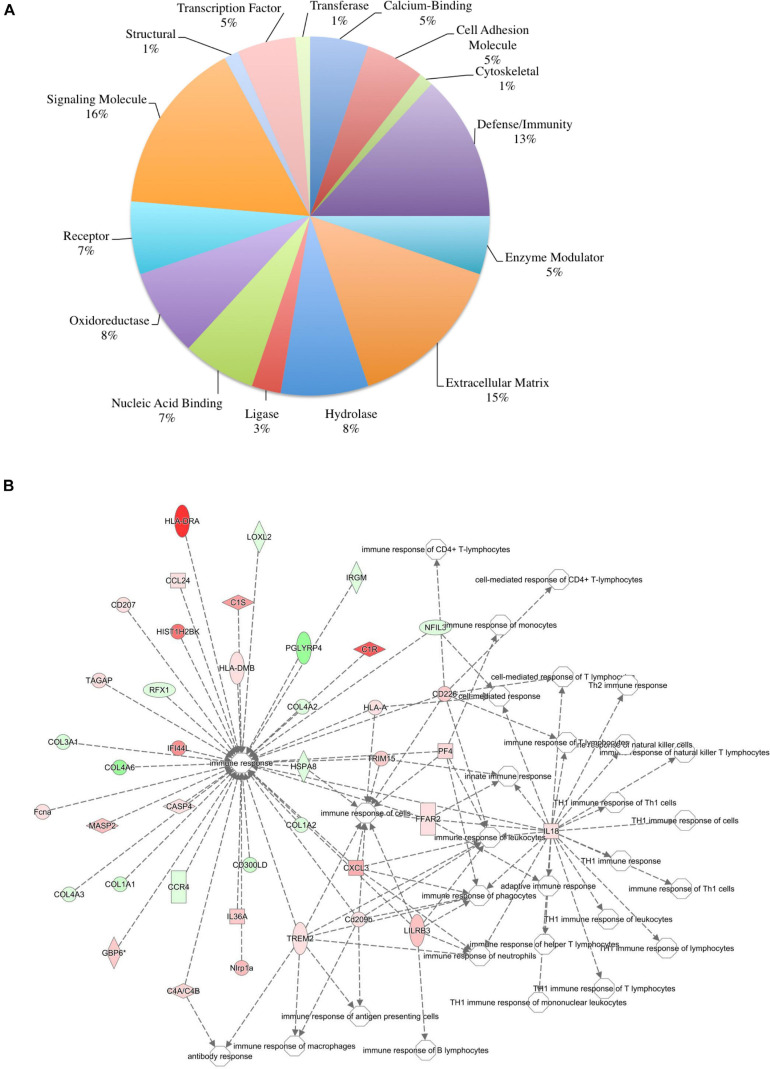
Overall immune response-associated transcriptional changes observed in BALB/c versus C57BL/6 normal skin. Total RNA was isolated from untreated BALB/c and C57 skin samples and subjected to transcriptome analysis by MiSeq. Differential gene lists were created with two-fold expression cutoff and significant FDR of <0.1 (*n* = 6/experimental group). **(A)** PANTHER protein class pie chart of all genes significantly modulated. **(B)** IPA network association of all genes significantly modulated with a known immune-associated function.

**FIGURE 5:**
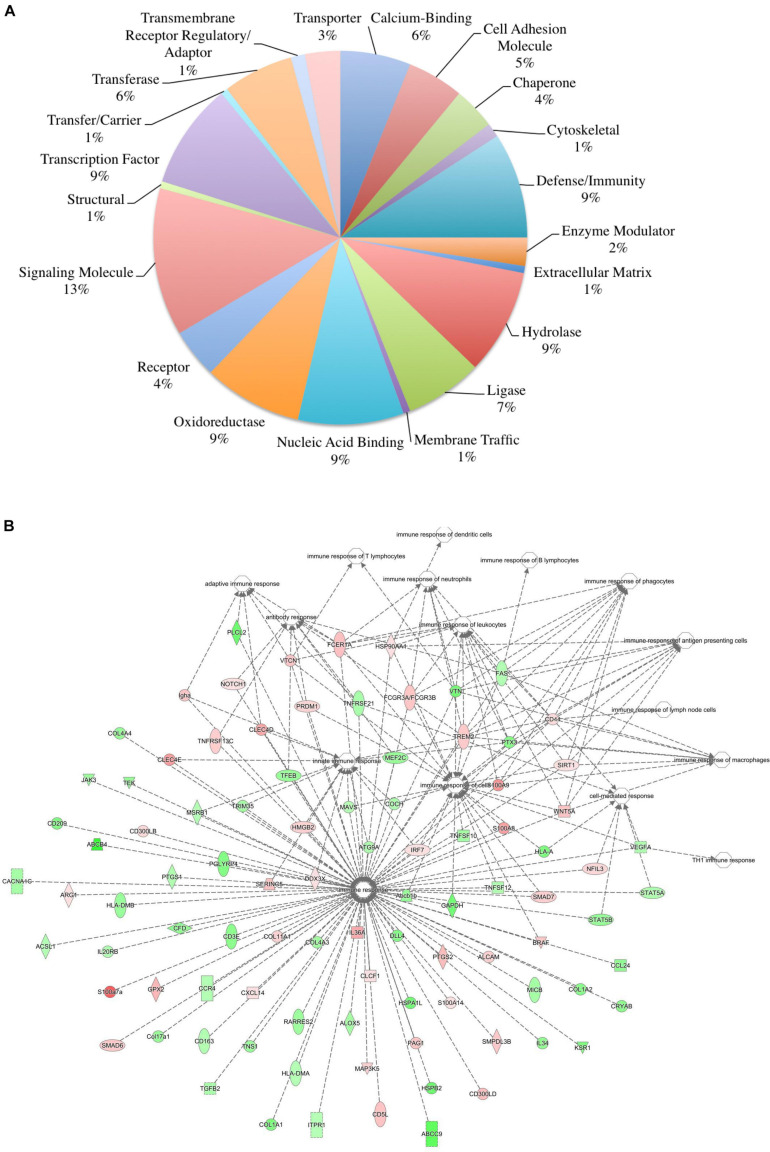
Overall immune response-associated transcriptional changes observed in C57BL/6 versus IL-6-deficient (KO) normal skin. Total RNA was isolated from untreated C57 and IL-6KO (KO) skin samples and subjected to transcriptome analysis by MiSeq (*n* = 6/experimental group). Differential gene lists were created with two-fold expression cutoff and significant FDR of <0.1. **(A)** PANTHER protein class pie chart of all genes significantly modulated. **(B)** IPA network association of all genes significantly modulated with a known immune-associated function.

To ensure epithelial integrity, the skin relies on innate defense mechanisms for rapid recognition and protection against invading pathogens. The cutaneous immune response requires the coordination of dermal and epidermal cells with the vast cytokine and chemokine network that is produced in addition to various immune cells (Salmon et al., 1994). Therefore, characterizing these three immunologically unique strains of mice was of primary interest, and further analysis was centered on genes associated with cutaneous immunity. Using various functional databases (IPA, DAVID, PANTHER, and CLC), over 220 genes were identified with an associated immune function where 69 were significantly different in BALB and 159 in KO as compared with C57 (Tables 1, 2). The associated PANTHER protein class, which is an extension of the PANTHER database (Mi et al., 2017) associated with a gene with a particular protein function that may not be included in the molecular function ontology, was then assessed. The distribution differed between strains: the majority of genes modulated in BALB were signaling or extracellular matrix associated, while the predominant protein class in IL-6KO skin was signaling molecules (Figure 4A vs. Figure 5A). Figures 4B, 5B further characterize the differentially immune-associated genes according to their associated function and reveal their relationship within an immunological network.

**TABLE 1:** Overall immune-associated transcriptional changes observed in BALB/c versus C57BL/6 normal skin.

Name	Log_2_ FC	FC	*p*-value	FDR *p*-value
**Upregulated**				
*H2-Ea-ps*	11.80	3,563.44	0.00	0.00
*C1rb*	9.69	826.18	0.00	0.00
*Hist1h2bk*	8.12	278.65	0.00	0.04
*Gbp2b*	6.64	99.86	0.00	0.00
*Ifi44l*	6.37	82.57	0.00	0.00
*C1s2*	5.58	47.90	0.00	0.00
*Cxcl2*	4.65	25.08	0.00	0.07
*Nlrp1a*	3.89	14.84	0.00	0.00
*Masp2*	3.66	12.65	0.00	0.01
Slc27a2*	3.49	11.22	0.00	0.00
9130204L05Rik*	3.47	11.08	0.00	0.01
*Lilra6*	3.42	10.74	0.00	0.00
*Il1f6*	3.05	8.29	0.00	0.00
*Cd226*	2.99	7.97	0.00	0.06
*Gbp10*	2.98	7.88	0.00	0.00
*Trim15*	2.95	7.74	0.00	0.03
*Gbp11*	2.70	6.50	0.00	0.00
Acsm3*	2.62	6.16	0.00	0.00
Krt6b*	2.56	5.90	0.00	0.00
*C4a*	2.48	5.58	0.00	0.00
5830411N06Rik*	2.26	4.78	0.00	0.08
*Pf4*	2.21	4.64	0.00	0.01
*Duox1*	2.06	4.17	0.00	0.00
*Tagap*	2.02	4.05	0.00	0.02
Cd163l1*	1.98	3.95	0.00	0.01
Col24a1*	1.62	3.07	0.00	0.00
Il1f10*	1.58	2.99	0.00	0.00
*Trem2*	1.56	2.95	0.00	0.01
*Ccl24*	1.55	2.93	0.00	0.00
Il1f8*	1.47	2.77	0.00	0.00
*Ffar2*	1.46	2.75	0.00	0.01
*H2-DMb2*	1.40	2.65	0.00	0.00
Cd209g*	1.38	2.59	0.00	0.00
Il1f9*	1.36	2.57	0.00	0.00
*H2-Q10*	1.28	2.43	0.00	0.01
Endou*	1.17	2.25	0.00	0.00
*Cd209b*	1.14	2.20	0.00	0.00
*Casp4*	1.12	2.17	0.00	0.04
*Cd207*	1.10	2.14	0.00	0.02
*Il18*	1.03	2.05	0.00	0.00
*Fcna*	1.02	2.02	0.00	0.04
**Downregulated**				
Trim12a*	−9.75	−859.88	0.00	0.01
*Col4a6*	−4.45	−21.83	0.00	0.00
*Pglyrp4*	−4.44	−21.72	0.00	0.00
Crnn*	−4.15	−17.73	0.00	0.00
Gm5849*	−2.97	−7.84	0.00	0.00
*Cd300ld*	−2.23	−4.71	0.00	0.02
S100a3*	−2.18	−4.54	0.00	0.04
*Col1a1*	−2.15	−4.45	0.00	0.00
Sectm1b*	−2.09	−4.25	0.00	0.02
Slamf7*	−1.89	−3.70	0.00	0.01
*Col3a1*	−1.70	−3.24	0.00	0.02
*Col1a2*	−1.61	−3.05	0.00	0.06
Creb3l1*	−1.58	−2.99	0.00	0.00
Mafa*	−1.54	−2.91	0.00	0.00
*Col4a3*	−1.45	−2.74	0.00	0.01
Col6a2*	−1.41	−2.65	0.00	0.00
Col6a1*	−1.36	−2.57	0.00	0.00
Loxl4*	−1.35	−2.55	0.00	0.01
Col5a1*	−1.34	−2.53	0.00	0.04
Col8a2*	−1.32	−2.49	0.00	0.00
*Loxl2*	−1.24	−2.37	0.00	0.02
*Irgm2*	−1.09	−2.12	0.00	0.00
Tril*	−1.08	−2.11	0.00	0.02
*Hspa8*	−1.07	−2.10	0.00	0.02
*Rfx1*	−1.05	−2.08	0.00	0.00
*Ccr4*	−1.04	−2.06	0.00	0.03
*Col4a2*	−1.02	−2.03	0.00	0.04
*Nfil3*	−1.01	−2.01	0.00	0.06

Differences were considered significant when FDR was <0.1 and fold change (FC) was >2.0. *Indicates genes that were not implemented into the IPA network in Figure 4B.

**TABLE 2:** Overall immune associated transcriptional changes observed in C57BL/6 versus IL-6-deficient (KO) normal skin.

Name	Log_2_ FC	FC	*p*-value	FDR *p*-value
**Upregulated**				
S100a3*	7.62	196.80	0.00	0.00
Crnn*	7.18	144.60	0.00	0.00
*Alox8*	6.19	73.09	0.00	0.00
*S100a7a*	5.98	62.96	0.00	0.00
Lef1*	5.10	34.36	0.00	0.00
*S100a9*	3.89	14.79	0.00	0.00
*Clec4e*	3.69	12.94	0.00	0.01
*S100a8*	3.50	11.28	0.00	0.00
Slc27a6*	3.34	10.13	0.00	0.00
*Clec4d*	3.23	9.40	0.00	0.00
*Il1f6*	3.20	9.20	0.00	0.00
Exo1*	3.08	8.45	0.00	0.00
*Ctps*	3.00	8.00	0.00	0.00
Styk1*	2.75	6.71	0.00	0.00
*Acsm3*	2.74	6.70	0.00	0.00
*9130204L05Rik*	2.65	6.29	0.00	0.01
*Gpx2*	2.64	6.25	0.00	0.00
*Ptgs2*	2.45	5.47	0.00	0.01
*Cd300ld*	2.40	5.28	0.00	0.00
*Krt6a*	2.37	5.18	0.00	0.00
*Fcer1a*	2.30	4.94	0.01	0.09
Pla2g4b*	2.26	4.80	0.00	0.00
*Serinc5*	2.26	4.80	0.00	0.00
Pla2g4c*	2.21	4.64	0.00	0.02
*Wnt5a*	2.19	4.56	0.00	0.00
*Cd5l*	2.17	4.51	0.00	0.04
*Igha*	2.17	4.49	0.01	0.09
*Vtcn1*	2.16	4.48	0.00	0.00
*Pag1*	2.13	4.38	0.00	0.00
*Braf*	2.09	4.26	0.00	0.00
*Fcgr4*	2.09	4.24	0.00	0.01
*Smad6*	2.05	4.14	0.00	0.00
*Smpdl3b*	1.99	3.97	0.00	0.00
Orai2*	1.98	3.95	0.00	0.00
*Trem2*	1.97	3.91	0.00	0.00
Aqp3*	1.91	3.75	0.00	0.00
Gm28042*	1.85	3.60	0.00	0.00
*Tnfrsf13c*	1.82	3.53	0.01	0.07
*Smad7*	1.82	3.53	0.00	0.00
Spint1*	1.82	3.52	0.00	0.00
Hmgb3*	1.79	3.45	0.00	0.00
*Alcam*	1.78	3.43	0.00	0.00
Il1f10*	1.75	3.37	0.00	0.00
*Map3k5*	1.74	3.35	0.00	0.00
*Col11a1*	1.70	3.24	0.00	0.00
Dtl*	1.67	3.18	0.00	0.00
*Hmgb2*	1.65	3.13	0.00	0.00
Efna2*	1.61	3.06	0.00	0.00
*Prdm1*	1.54	2.90	0.00	0.00
*Nfil3*	1.52	2.86	0.00	0.00
Enpp1*	1.50	2.82	0.00	0.00
Runx2*	1.42	2.68	0.00	0.00
*Cd300lb*	1.39	2.62	0.01	0.07
*Cd44*	1.37	2.58	0.00	0.00
Acsl3*	1.35	2.55	0.00	0.00
Rnf19b*	1.34	2.53	0.00	0.00
S100a16*	1.33	2.52	0.00	0.00
Nradd*	1.24	2.37	0.00	0.01
Cad*	1.15	2.22	0.00	0.00
*Notch1*	1.14	2.20	0.00	0.01
*Irf7*	1.11	2.17	0.00	0.02
*Cxcl14*	1.09	2.13	0.00	0.00
*Clcf1*	1.09	2.12	0.00	0.06
*Sirt1*	1.08	2.11	0.00	0.01
Nlk*	1.08	2.11	0.00	0.01
*Ddx3x*	1.07	2.10	0.00	0.04
*Tbx1*	1.06	2.09	0.00	0.01
*Hsp90aa1*	1.04	2.06	0.00	0.01
*S100a14*	1.02	2.03	0.01	0.10
*Arg1*	1.01	2.02	0.00	0.01
**Downregulated**				
Col22a1*	−3.08	−8.48	0.00	0.00
*Wfikkn2*	−2.85	−7.19	0.00	0.00
*Abcb4*	−2.82	−7.08	0.00	0.00
Acsm5*	−2.81	−7.00	0.00	0.05
Prkag3*	−2.74	−6.68	0.00	0.00
Txlnb*	−2.40	−5.29	0.00	0.00
Hspb3*	−2.26	−4.80	0.00	0.00
*Abcc9*	−2.20	−4.60	0.00	0.00
Carmil3*	−2.14	−4.40	0.00	0.04
Pxmp2*	−2.01	−4.02	0.00	0.00
*Hspb2*	−2.00	−4.01	0.00	0.00
*Srpk3*	−2.00	−3.99	0.00	0.00
*Plcl2*	−1.97	−3.91	0.00	0.00
*Hspb6*	−1.96	−3.88	0.00	0.00
*Vtn*	−1.93	−3.82	0.00	0.00
*Hspa1l*	−1.92	−3.78	0.00	0.00
*Gapdh*	−1.90	−3.74	0.00	0.00
Gm5849*	−1.85	−3.59	0.00	0.06
*Col1a1*	−1.78	−3.44	0.00	0.04
Bcl6b*	−1.78	−3.44	0.00	0.01
*H2-Q7*	−1.76	−3.39	0.00	0.00
Col8a2*	−1.75	−3.36	0.00	0.00
Mal*	−1.74	−3.33	0.00	0.00
*Cd209e*	−1.73	−3.32	0.01	0.09
Fhl1*	−1.73	−3.31	0.00	0.00
Acsl6*	−1.73	−3.31	0.00	0.00
*Ksr1*	−1.73	−3.31	0.00	0.00
*C7 (cxcl10)*	−1.67	−3.18	0.00	0.02
*Pglyrp4*	−1.66	−3.17	0.00	0.00
*Ptx3*	−1.66	−3.15	0.01	0.09
*Dll4*	−1.64	−3.11	0.00	0.00
Gck*	−1.62	−3.07	0.00	0.01
*Col1a2*	−1.59	−3.01	0.01	0.08
Susd2*	−1.57	−2.97	0.00	0.00
*Cryab*	−1.57	−2.96	0.00	0.00
*Cd3e*	−1.56	−2.94	0.00	0.03
Gimap6*	−1.55	−2.93	0.00	0.00
Hspb7*	−1.54	−2.91	0.00	0.00
*Cfd*	−1.50	−2.83	0.00	0.05
*Ccl24*	−1.50	−2.83	0.00	0.01
Mapk12*	−1.49	−2.81	0.00	0.00
*Il34*	−1.49	−2.81	0.00	0.00
Tbkbp1*	−1.46	−2.76	0.00	0.00
*Slc27a1*	−1.46	−2.75	0.00	0.00
Sectm1b*	−1.45	−2.73	0.01	0.08
*Col4a4*	−1.44	−2.71	0.00	0.00
*Col17a1*	−1.43	−2.70	0.00	0.03
*Stat5b*	−1.43	−2.70	0.00	0.00
*Mef2c*	−1.43	−2.69	0.00	0.00
*Tfeb*	−1.42	−2.68	0.00	0.00
*Abcb1b*	−1.41	−2.66	0.00	0.00
Tns2*	−1.41	−2.65	0.00	0.00
*Tek*	−1.41	−2.65	0.00	0.00
*Tns1*	−1.37	−2.59	0.00	0.00
*H2-DMb2*	−1.37	−2.59	0.01	0.08
Sectm1a*	−1.36	−2.56	0.00	0.02
*Rarres2*	−1.34	−2.53	0.00	0.00
*Jak3*	−1.34	−2.52	0.00	0.00
Tst*	−1.33	−2.51	0.00	0.00
Acss1*	−1.32	−2.50	0.00	0.00
*Col4a3*	−1.31	−2.49	0.00	0.04
*Mgl2*	−1.31	−2.47	0.00	0.01
Gpx3*	−1.28	−2.43	0.00	0.04
Peli2*	−1.27	−2.41	0.00	0.00
*Tnfsf10*	−1.26	−2.39	0.00	0.01
*Trim35*	−1.25	−2.37	0.00	0.00
*Alox5*	−1.22	−2.33	0.00	0.03
*Mill2*	−1.22	−2.33	0.00	0.02
*Cacna1c*	−1.21	−2.32	0.00	0.05
*Tgfb2*	−1.19	−2.28	0.00	0.00
*Tnfrsf21*	−1.17	−2.25	0.00	0.00
*Atg9a*	−1.16	−2.24	0.00	0.00
Tnk1*	−1.15	−2.22	0.00	0.01
Ppp2r2c*	−1.13	−2.19	0.01	0.09
*Coch*	−1.11	−2.16	0.00	0.01
*Vegfa*	−1.09	−2.13	0.00	0.01
Abcc5*	−1.09	−2.13	0.00	0.00
*Stat5a*	−1.08	−2.12	0.00	0.00
*Tnfsf12*	−1.08	−2.11	0.00	0.02
*Il20rb*	−1.08	−2.11	0.00	0.04
*Ptgs1*	−1.07	−2.10	0.00	0.06
*Cd163*	−1.06	−2.08	0.00	0.05
*Fas*	−1.05	−2.07	0.01	0.08
*Ccr4*	−1.04	−2.06	0.00	0.05
*Acsl1*	−1.04	−2.05	0.01	0.08
*Msrb1*	−1.02	−2.03	0.00	0.00
*Mavs*	−1.02	−2.03	0.00	0.00
*H2-DMa*	−1.01	−2.01	0.00	0.02
*Itpr1*	−1.00	−2.00	0.00	0.00

Differences were considered significant when FDR was <0.1 and fold change (FC) was >2.0. *Indicates genes that were not implemented into the IPA network in Figure 5B.

### Taxonomical Distribution of Bacterial Populations and Altered Expression of Antimicrobial Associated Genes in BALB/c, C57BL/6, and IL-6KO Mice

**FIGURE 6:**
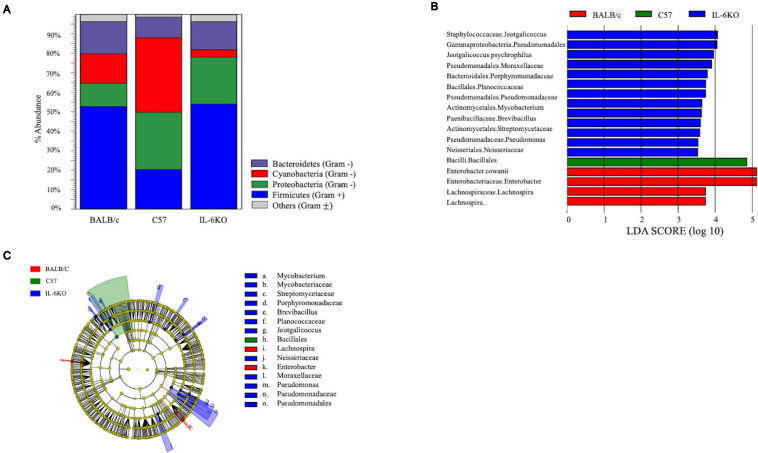
Microbial community composition is altered in skin from various immunologically predisposed mouse strains. **(A)** 16S rRNA sequencing was performed on BALB/c, C57BL/6, and IL-6-deficient (KO) skin samples, and relative abundance of bacterial present on the skin is presented (*n* = 6/experimental group). Taxonomy presented at the phylum level. **(B)** Linear discriminant effect size analysis (LEfSe) of the microbial communities showed significant (*p* < 0.05) differences between the C57 and either BALB/c or the IL-6KO skin microbiomes with over 70% of the statistically relevant changes attributed to the altered IL-6KO community. Taxonomy is presented at the family level where known. The complete taxonomy and associated table are presented in Supplementary Figures 2, 3, and Supplementary Table 1. **(C)** The relevant differences in microbial communities are mapped upon a cladogram created using the LEfSe analysis. The largest number of statistically altered communities is mapped to the population associated with the IL-6KO (KO) (*p* < 0.05).

The skin supports and promotes a symbiotic relationship with a vast number of bacterial communities, which can ultimately serve to protect the host from pathogenic bacteria. Environmental factors, host immunity, and organism adherence and virulence are intricately related to cutaneous infection. As indicated in Figures 4, 5, the genetic background of the host can influence the transcriptional profile of the immune response; however, much less research has focused on the skin immune phenotype and how this ultimately influences microbial colonization. Therefore, 16S ribosomal sequencing was performed on normal skin from each strain (BALB, accession number SAMN17494078; C57, accession number SAMN17494079; KO, accession number SAMN17494080). There were dissimilar bacterial communities at the phylum and genus levels for all three strains (Figure 6 and Supplementary Figure 2). It is shown that the majority of bacteria that colonized BALB normal skin fall within the phylum Firmicutes (52%), followed by Bacteroidetes (16%), Cyanobacteria (15%), and Proteobacteria (12%). C57 normal skin was predominantly colonized by Cyanobacteria (38%) and Proteobacteria (30%), with significantly decreased presence of Firmicutes (21%) as compared with BALB/c and KO. Remarkably, deleting a single gene (IL-6) from C57 changed the skin microbiome dramatically as compared with the control, where the phyla Firmicutes (54%), Proteobacteria (24%), and Bacteroidetes (12%) were present. Additionally, KO skin had a significantly higher bacterial load as compared with C57 and BALB as indicated by the overall abundance (data not shown, 3.3203E + 6 vs. 1.1914E + 06 vs. 652,858). LEfSe analysis identifies features most likely to explain the differences between groups with features such as organisms, clades, genes, operational taxonomic units, or functions (Segata et al., 2011). LEfSe was performed to determine which taxa are statistically significant (*p* < 0.5) between the three populations, and a total of 18 taxa were identified as statistically significant (Figure 6B). It was shown that IL-6-deficient mice have the most significant changes to the microbiome, with over 70% of the statistically relevant taxa attributed to this treatment group followed by BALB/c and C57. A cladogram presenting these results is shown in Figure 6C.

In addition, the skin is constantly challenged by potential pathogens but is rarely infected unless other complications occur. One critical mechanism the skin initiates to defend against potentially invading microorganisms is the production of antimicrobial lipids and proteins, proteases, and reactive oxygen species (Belkaid and Segre, 2014). Unlike in the gut, the role of microbes on the surface of the skin and the release of these peptides have not been well documented. Therefore, genes associated with antibacterial response were selected using the Qiagen Ingenuity Pathway Analysis (IPA) software as well the functional annotation database, DAVID, to categorize significantly modulated genes. It was found that the expressions of 91 genes with functions associated with antibacterial response were significantly modulated in BALB and KO as compared with C57. The majority of genes modulated in BALB skin were significantly upregulated with phospholipase A2 Group IIA (*Pla2g2a*), histone cluster 1 H2B Family Member K (*Hist1h2bk*), histone cluster 1 H2A Family Member L (*Hist1h2al*), guanylate-binding protein 2b (*Gbp2b*), and C-X-C motif chemokine ligand 2 (*CXCL2*) over 20-fold higher than C57, while ribosomal protein L29 (*Rpl29*), adenosylhomocysteinase (*Ahcy*), hepcidin antimicrobial peptide 2 (*Hamp2*), and peptidoglycan recognition protein 4 (*Pglyrp4*) were 20-fold lower (Figure 3). Interestingly, the five most upregulated genes [lysozyme G2 (*Lyg2*), S100 calcium-binding protein A3 (*S100a3*), *CXCL2*, keratin 6b (*Krt6b*), aconitate decarboxylase 1 (*Acod1*)] in KO skin were over 100-fold higher than the control C57 skin. IL-22ra1, stabilin 2 (*Stab2*), *Hamp2*, laminin subunit alpha 1 (*Lama1*), and nitric oxide synthases 2 (*Nos2*) were all downregulated by at least five-fold in KO (Tables 3, 4).

**TABLE 3:** Antimicrobial associated genes differentially modulated in BALB/c skin as compared with C57BL/6 skin.

Name	Log_2_ FC	FC	*p*-value	FDR *p*-value
**Upregulated**				
*Pla2g2a*	8.62	394.41	0.00	0.00
*Hist1h2bk*	8.12	278.65	0.00	0.04
*Hist1h2al*	7.52	183.45	0.00	0.08
*Gbp2b*	6.64	99.86	0.00	0.00
*Cxcl2*	4.65	25.08	0.00	0.07
*Baiap2l1*	4.47	22.15	0.00	0.00
*Defb14*	3.95	15.46	0.00	0.00
*Nlrp1a*	3.89	14.84	0.00	0.00
*Gbp10*	2.98	7.88	0.00	0.00
*Krt6b*	2.56	5.90	0.00	0.00
*Chga*	2.40	5.26	0.00	0.05
*H2-K2*	2.39	5.25	0.00	0.00
*Pla2g2d*	1.91	3.77	0.00	0.00
*Alpk1*	1.64	3.11	0.00	0.00
*Lyz1*	1.48	2.80	0.00	0.00
*Serpine1*	1.37	2.58	0.00	0.00
*Cd209b*	1.14	2.20	0.00	0.00
*Casp4*	1.12	2.17	0.00	0.04
**Downregulated**				
*Irgm2*	−1.09	−2.12	0.00	0.00
*Rpl30*	−1.16	−2.24	0.00	0.00
*Coch*	−1.37	−2.58	0.00	0.00
*Vwf*	−1.53	−2.88	0.00	0.00
*S100a3*	−2.18	−4.54	0.00	0.04
*Thbs4*	−2.67	−6.34	0.00	0.00
*Lama1*	−2.89	−7.40	0.00	0.00
*Ccl19*	−2.99	−7.93	0.00	0.00
*Trim5*	−3.35	−10.22	0.00	0.01
*Pglyrp4*	−4.44	−21.72	0.00	0.00
*Hamp2*	−4.64	−24.94	0.00	0.01
*Ahcy*	−4.92	−30.37	0.00	0.00
*Rpl29*	−7.47	−177.74	0.00	0.00

*Using the statistical cutoff described (FC > 2, FDR < 0.1), genes modulated in the pairwise comparison in BALB/c versus C57 normal skin with an associated antimicrobial activity were then selected for using various databases or software (DAVID, PANTHER, IPA, and CLC) and presented.*

**TABLE 4:** Antimicrobial associated genes differentially modulated in IL-6-deficient (KO) skin as compared with C57BL/6 skin.

Name	Log_2_ FC	FC	*p*-value	FDR *p*-value
**Upregulated**				
*Lyg2*	7.70	207.70	0.00	0.00
*S100a3*	7.62	196.80	0.00	0.00
*Cxcl2*	7.07	134.53	0.00	0.00
*Krt6b*	6.79	110.64	0.00	0.00
*Acod1*	6.69	103.27	0.00	0.05
*Il1b*	5.28	38.74	0.00	0.00
*Trem1*	4.59	24.03	0.00	0.01
*Cxcr2*	4.57	23.83	0.00	0.00
*Saa3*	3.99	15.89	0.00	0.00
*S100a9*	3.89	14.79	0.00	0.00
*Slpi*	3.74	13.35	0.00	0.00
*Slpi*	3.74	13.35	0.00	0.00
*Clec4e*	3.69	12.94	0.00	0.01
*Il6*	3.61	12.18	0.00	0.01
*S100a8*	3.50	11.28	0.00	0.00
*Igkc*	3.47	11.11	0.00	0.01
*Clec4d*	3.23	9.40	0.00	0.00
*Gpx2*	2.64	6.25	0.00	0.00
*Wfdc12*	2.58	5.96	0.00	0.00
*Krt6a*	2.37	5.18	0.00	0.00
*Fzd5*	2.20	4.60	0.00	0.00
*Wnt5a*	2.19	4.56	0.00	0.00
*Igha*	2.17	4.49	0.01	0.09
*Defb14*	2.16	4.48	0.01	0.08
*Adamts4*	1.90	3.72	0.00	0.01
*Hmgb2*	1.65	3.13	0.00	0.00
*Epha2*	1.47	2.76	0.00	0.00
*Serpine1*	1.37	2.58	0.00	0.04
*Lyst*	1.26	2.39	0.00	0.00
*Ahcy*	1.23	2.34	0.00	0.00
*Cad*	1.15	2.22	0.00	0.00
*Cxcl14*	1.09	2.13	0.00	0.00
**Downregulated**				
*Mavs*	−1.02	−2.03	0.00	0.00
*Ccr4*	−1.04	−2.06	0.00	0.05
*Fas*	−1.05	−2.07	0.01	0.08
*Syt11*	−1.08	−2.11	0.01	0.07
*Coch*	−1.11	−2.16	0.00	0.01
*Tnfrsf21*	−1.17	−2.25	0.00	0.00
*Il6ra*	−1.18	−2.26	0.00	0.01
*Peli2*	−1.27	−2.41	0.00	0.00
*Adh7*	−1.28	−2.43	0.00	0.00
*Rarres2*	−1.34	−2.53	0.00	0.00
*Ccl19*	−1.36	−2.56	0.01	0.07
*Nr1h3*	−1.37	−2.59	0.00	0.00
*Tfeb*	−1.42	−2.68	0.00	0.00
*Mef2c*	−1.43	−2.69	0.00	0.00
*Stat5b*	−1.43	−2.70	0.00	0.00
*H2-K2*	−1.52	−2.86	0.01	0.08
*Vwf*	−1.53	−2.89	0.00	0.00
*Il18r1*	−1.54	−2.91	0.00	0.06
*Pglyrp4*	−1.66	−3.17	0.00	0.00
*Mal*	−1.74	−3.33	0.00	0.00
*Ahrr*	−1.83	−3.56	0.00	0.07
*Vtn*	−1.93	−3.82	0.00	0.00
*Mbp*	−2.21	−4.64	0.00	0.00
*Nos2*	−2.48	−5.60	0.00	0.00
*Lama1*	−2.49	−5.60	0.00	0.00
*Hamp2*	−2.61	−6.12	0.00	0.06
*Stab2*	−2.81	−7.01	0.00	0.01
*Il22ra2*	−2.88	−7.37	0.00	0.00

*Using the statistical cutoff described (FC > 2, FDR < 0.1), genes modulated in the pairwise comparison in IL-6KO versus C57 normal skin with an associated antimicrobial activity were then selected for using various databases or software (DAVID, PANTHER, IPA, and CLC) and presented.*

## Discussion

We recently identified significant transcriptional changes between two different mouse strains using an irritant contact dermatitis mouse model in which normal skin was exposed to benzalkonium chloride (BKC) (Luckett-Chastain et al., 2018). While understanding the mechanism of cutaneous disease states is a high priority, further characterizing the transcriptional and microbe–genetic interaction in immunologically diverse mouse strains is another fundamental concept that has yet to be fully elucidated. Although there have been great strides made in translational research concerning cutaneous wound repair, discrepancies between human and murine skin repair models still remain. Murine skin does not mirror that of human skin with morphological, physiological, and immunological differences apparent (Zomer and Trentin, 2018). Additionally, the use of larger mammals that are physiologically closer to humans, such as pigs, has increased in translational studies (Lindblad, 2008); however, pigs are not as well characterized at the cellular and physiological levels when compared with mice, and specific swine reagents, such as antibodies and growth factors, are still not available. Despite these interspecies differences, the murine model is still the most commonly accepted and used model of repair with care taken when interpreting results to highlight any of these particularities. Therefore, in order to better understand the influence of genetic background on homeostatic skin gene and bacterial expression, a comprehensive genetic analysis was performed using high-throughput sequencing of RNA isolated from the skin from C57BL/6 (control), BALB/c varied at a polygenic level, and IL-6 deficient (KO) varied by a single gene deletion. As previously stated, C57BL/6 and BALB/c mice are respectively regarded as Th1 and Th2 immune response dominant mouse strains, while IL-6 is a well-known pleiotropic cytokine that plays various roles in overall immunity and cutaneous repair, as well as in homeostasis. The Th1/Th2 hypothesis emerged in the late 1980s, stemming from observations in mice of two subtypes of T-helper cells differing in cytokine secretion patterns and other functions. However, many lines of evidence highlight the fact that this model is overly simplistic. Indeed, it has been shown that the anatomy of the skin (albeit through deletion of the IL-6R), ICD severity, and repair mechanisms between these models vary. For example, histologically, KOs have a bit thinner skin with altered stratum corneum, whereas C57 and BALB skin looks quite similar, but inflammation varies significantly between them. Furthermore, C57BL/6 develop more a severe ICD after irritant exposure as compared with BALB/c, while deletion of IL-6 further exacerbates the inflammatory process in ICD (Lee et al., 2013; Calhoun et al., 2019; Frempah et al., 2019). Additionally, wound repair differs between mouse strains with BALB/c mice healing slower than C57BL/6 and KO even having a further delay in repair (Gallucci et al., 2000; Pal-Ghosh et al., 2008). Despite the varying mechanisms and views regarding strains, there has yet to be a comprehensive study investigating the transcriptional differences in the skin relative to the strain, as well as what effect the systemic loss of a keystone cytokine such as IL-6 will have, making this study novel in nature.

### Overall Analysis

The first analysis was to examine the global changes seen in BALB and KO skin as compared with C57. Interestingly, KO skin displayed almost six times more genes variably modulated than BALB/c when compared with C57 (2,686 vs. 461, Figure 1) with the majority of genes falling within the −3- to 3-fold change difference range (Figure 3) and associated with metabolic and cellular processes (Supplementary Figure 1). While there is a well-established role for IL-6 in systemic metabolic control (Covarrubias and Horng, 2014), little is known concerning IL-6 in skin metabolism. Even though the skin is the major protective barrier against the environment, it should not only be thought of as merely an inert organ, but also as a chemically active barrier, with enzymes located in the viable epidermis as well as in the extracellular spaces of the stratum corneum and the dermis. Metabolic processes in the skin may increase the water solubility of foreign substances to achieve an increased excretion and elimination rate. Interestingly, with respect to the number of cells, the skin possesses a biotransformation activity of one-third that of the liver and can ultimately influence the efficacy of dermally applied drugs. Furthermore, skin metabolism ultimately controls how immune and skin cells respond to topical xenobiotics and may play a role in the manifestation or amelioration of adverse effects. For example, the two main barrier functions of the skin that are imperative for survival are the permeability and antimicrobial barriers, where lipids play a huge role in mediating these barrier functions. Interestingly, alterations in lipid homeostasis by commensal bacteria can alter immune system function by establishing a metabolic state that favors some immune processes over others (Brestoff and Artis, 2013). For example, T-cell differentiation into effector or memory cells relies on fatty acid oxidation at steady state (Wang and Green, 2012). Th1, Th2, and Th17 cells rely upon glucose metabolism, whereas regulatory T cells and memory CD8^+^ T cells exhibit increased fatty acid oxidation (Pearce et al., 2009). Inhibition of glycolysis alters the development of Th17 cells and promotes the generation of T regulatory cells (Shi et al., 2011) influencing the direction of the immune response.

Additionally, keratinocytes and skin immune cells are continually metabolizing nutrients present in their microenvironment. Of note, IL-6KO skin expressed over 900 genes associated with metabolic processes, while BALB/c only expressed around 200. Interestingly, single-nucleotide polymorphisms (SNPs) in the promoter region of *IL-6* (rs1800797) have been linked to increased risk of diabetes and several metabolic syndrome phenotypes (Hamid et al., 2005; Huth et al., 2006; Shen et al., 2008), further supporting a role for IL-6 in skin metabolic processes.

### Immune Function

The skin is a prototypical organ involved in not only mechanical but also immunological barriers to pathogens as well as in body homeostasis (Salmon et al., 1994; Quaresma et al., 2017; Quaresma, 2019). The dermis contains most of the lymphocytes in the skin including migrant leukocytes, mast cells, and resident tissue macrophages, and although the epidermis does not have access to the circulating lymphatics, it does play host to other immune-competent cells (Salmon et al., 1994). As such, the overall immune gene expression differences between the three mouse strains were also investigated. One of the genes most significantly upregulated in BALB/c normal skin was the complement component *C1rb* (826.17-fold) with additional members of the compliment family, *C1s2*, *Masp2*, and *C4a*, also significantly increased. Interestingly, complement dysregulation, deficiency, and genetic polymorphisms have been associated with a number of cutaneous diseases, such as psoriasis, atopic dermatitis, SLE, pemphigus vulgaris, bullous pemphigoid, and reoccurring cutaneous infections (Chehoud et al., 2013). Similarly, it was shown that the *Cr1b* homolog, *Cr1*, was significantly expressed in conventionally raised mice as compared with germ-free mice suggesting that the commensal microbiota has a role in positively regulating the expression of genes encoding complement components (Chehoud et al., 2013). Other immune-regulatory genes such as guanylate-binding protein *Gbp2b*, which plays an active role in the regulation of inflammasomes (Sohrabi et al., 2018); histocompatibility 2, class II antigen E alpha (*H2-Ea-ps*) and histocompatibility 2, Q region locus 2 (*H2-Q2*), both of which are involved in antigen presentation; and the neutrophil chemoattractant, *Cxcl2*, were also significantly increased (Figure 6). Together, this may represent an arsenal of immune surveillance modulators that are upregulated to maintain a state of homeostasis, to ward off pathogens, and may account for the decreased bacterial load of BALB/c skin.

Conversely, the signature of genes differentially decreased in BALB/c compared with control was varied. *Trim12a* and *Pglyrp4* were highly downregulated (860–20-fold reduction) in BALB/c skin (Table 1). *Trim12a* is a member of the tripartite motif-containing superfamily of the E3 ubiquitin ligases, expressed in response to type I and II interferons (Ozato et al., 2008) and involved in a broad range of biological processes that are associated with innate immunity (Reymond et al., 2001; Rajsbaum et al., 2008). *Pglyrp4*, which has been reported to influence the innate and adaptive immunity as well as atopic dermatitis through modulation of Treg versus Th17 activity. Additionally, numerous members of the collagen family (*Col4a6*, *Col1a1*, *3a1*, *1a*2, *4a3*, *6a1*, *5a1*, and *8a2*); *Crnn*, a marker of keratinocyte differentiation; and *S100a3*, a calcium-binding protein involved in regeneration, also decreased. The quality and quantity of collagen in the skin is affected by both endogenous and exogenous factors, including disease, hormones, stress, aging, and treatment with topicals. Decreased expression of this gene signature in BALB/c samples may highlight the anatomical differences in BALB/c that could potentially contribute to altered healing and inflammatory conditions seen in a more Th2-weighted immune system (Watanabe et al., 2004).

The epidermal barrier is the first line of defense against skin injury and any invading pathogens. Keratinocytes are a major source of inhibitory cytokines allowing the skin to remain in inflammatory quiescence or homeostasis. Any perturbation of the balance between the pro- and anti-inflammatory signals (such as the loss of IL-6) may lead to the development of chronic inflammatory conditions such as psoriasis or atopic dermatitis (Salmon et al., 1994; Albanesi et al., 2007). As such, one would anticipate the loss of IL-6 to modulate a more diverse transcriptional signature as compared with BALB/c and control. The most commonly represented family of genes upregulated in KO skin was the calcium-binding cytosolic proteins S100s (*S100a3*, *S100a7a*, *S100a9*, *S100a8*). This multigene family has a broad range of intracellular and extracellular functions through regulating calcium balance, cell apoptosis, migration, proliferation, differentiation, energy metabolism, and inflammation and maintaining immunological homeostasis (Donato et al., 2013; Xia et al., 2017), Elevated expression of various S100 proteins have been associated with psoriasis, impaired wound healing, atopic dermatitis (Xia et al., 2017), tumors, and obesity (Donato et al., 2013). Interestingly, *Crnn* (Li et al., 2019), *Alox8* (Schneider et al., 2004), *Lef11* (Steinke and Xue, 2014), *Clec4e*/d (Patin et al., 2017), and *Gpx2* (Walshe et al., 2007), all of which have known protective or compensatory mechanisms for inflammation, were also shown to be upregulated. We have previously indicated the anti-inflammatory mechanism associated with IL-6 in an irritant contact dermatitis model (Lee et al., 2013). These data may further indicate that in the absence of IL-6, additional mediators are initiated in order to compensate for the loss of IL-6 and maintain homeostatic conditions in the skin.

Interestingly, many of the most significantly downregulated genes in KO skin were genes with unknown functions associated with cutaneous immunity and may be downregulated directly by IL-6 as it has a plethora of associated functions. This may provide novel targets to investigate in further IL-6-related studies but is beyond the scope of this manuscript.

### Antimicrobial Proteins and Bacterial Colonization

In addition to being a physical barrier against foreign pathogens, the skin concomitantly provides a home to a myriad of commensal bacteria and serves as an immunological barrier. The capacity of a microorganism, including “healthy” microbiota, to promote disease or infection depends upon factors such as the state of the host’s immune system, genetic predisposition, and location (Belkaid and Segre, 2014; Belkaid and Tamoutounour, 2016). Additionally, the pathogen has to overcome the numerous layers of the innate immune mediators such as antibacterial peptides, proteases, reactive oxygen species, and other proteins induced to provide surveillance and recognition of foreign objects (Belkaid and Tamoutounour, 2016). In this study, it was shown that even in normal skin, components of the innate defense mechanism are modulated and vary depending on the strain of mouse (Tables 3, 4 and Figure 6).

While there is some redundancy in antimicrobial associated genes between strains, it is interesting to note that there is also a unique expression signature within each strain, for example, the modulation of phospholipase A2 Group IIA (*Pla2g2a*) and phospholipase A2 Group IID (*Pla2g2d*) and tripartite motif containing 5 (*Trim5*) in BALB/c skin. *Pla2g2d* also known as the “resolving sPLA2” ameliorates inflammation through mobilizing proresolving lipid mediators and reducing Th1 cytokine production (Miki et al., 2013), while *Pla2g2a* can be stimulated by IL-6 production and protects against Gram-positive bacterial infection (Nevalainen et al., 2008). Additionally, the knockdown of Trim5 has been shown to decrease inflammatory production of *CXCL10*, *IL-8*, and *CCL8* (Pertel et al., 2011). Therefore, with the downregulation of *Trim5*, along with *Pla2g2a* and *Pla2g2d* upregulation in BALB/c skin, a more Th2-driven, homeostatic environment may potentially be more favored (Figure 4A).

Although the bacterial composition was similar between BALB/c and IL-6-deficient strains, the antimicrobial response varied between the two. *Lyg2*, which encodes proteins that bind LPS and peptidoglycan, was almost 200× greater than C57 control and has been shown to inhibit the growth of Gram-positive and not Gram-negative. This elevated expression, along with the increased expression of *S100a3*, *CXCL2*, aconitate decarboxylase1 (*Acod1*), serum amyloid A3 (*Saa3*), *IL-1*β, triggering receptor expressed on myeloid cells 1 (*Trem1*), and *CXCR2* (which is the CXCL2 receptor), may be a pro-inflammatory mechanism activated in order to keep the bacterial load in check in the absence of IL-6 (Lagler et al., 2009; Sahoo et al., 2011; Zenobia et al., 2013; Luan and Medzhitov, 2016). This can be supported by our previous study that indicates IL-6KO mice have an exacerbated inflammatory response when exposed to chemical irritants (Lee et al., 2013). Furthermore, children that possess autoantibodies against IL-6 develop recurrent staphylococcal cellulitis and subcutaneous abscesses (Puel et al., 2008) and IL-6KO did demonstrate a higher presence of *Staphylococcus* as seen in Figure 6B.

Defining the “normal” skin microbiome has become quite challenging due to the dynamic physical and chemical features of the skin. Furthermore, the population of organisms comprising the microbiome is mostly commensal and represents greater collective metabolic capacity than the human host. The skin has the ability to provide various environmental niches based on anatomical location that vary greatly depending on humidity, temperature, oiliness, and aerobicity, all of which can influence the growth of resident microbials and result in a diverse skin-resident bacterial presence. As previously defined, most skin bacteria fall into four different phyla: Actinobacteria (being the most dominant), Firmicutes, Bacteroidetes, and Proteobacteria with the proportions varying between all three strains seen in Figure 6A. As compared with C57 control, IL-6KO and BALB/c were primarily colonized by members of the Gram-positive phylum Firmicutes, which includes members of the well-known genera *Streptococcus* and *Staphylococcus*. Analysis of the human skin microbiome has demonstrated the presence of photosynthetic cyanobacteria, especially in areas receiving sunlight (Cho and Blaser, 2012), especially on the crown of the head and tip of the nose (Grice and Segre, 2011). Thus, it is no surprise that such microbes also colonize mouse skin, and indeed, it was found that Cyanobacteria was the predominate phylum of bacteria in C57 control mice. While very few studies have addressed an association with skin, cyanobacteria, and disease, a study by Pilotto et al. (2004) did find some species of Cyanobacteria that resulted in acute irritant effects upon skin exposure. Similarly, the mouse model of keratitis has been characterized extensively and shown that different strains of mice respond to infection with differing cytokine profiles. More specifically, mice favoring a Th1 response (C57) were more susceptible to bacterial infection compared with mice favoring a Th2 response (BALB/c) (Wynants et al., 2000).

Interestingly, the overall bacterial load on KO skin samples was significantly higher than the other two strains indicating a potential lack of bacterial clearance, or perhaps the lack of IL-6 promotes a more conducive environment for bacterial colonization (data not shown). This finding is supported by a recent study in which mice lacking IL-6 failed to control bacterial numbers 2–3 weeks after infection with *Citrobacter rodentium* (Dann et al., 2008), controlled excessive inflammation (Hume et al., 2006), and protected the cornea during *Pseudomonas aeruginosa* infection (Cole et al., 1999). Furthermore, in a model of pneumococcal pneumonia, IL-6^–/–^ mice showed six times more pneumococci in the lung tissue and a higher mortality than wild-type mice (van der Poll et al., 1997; Bergeron et al., 1998). Additionally, patients with mutations within the gene encoding STAT3, an IL-6-induced transcription factor, show impaired IL-6 activity and were susceptible to recurring Gram-negative infections of the skin, lung, and gut (Freeman and Holland, 2010). This was further supported by the fact that IL-6 plays a crucial role in the inflammatory response to infectious agents such as Gram-negative bacteria (Dalrymple et al., 1995). However, such interactions between the skin microbiome and IL-6 function may well be a two-way street as a recent study showed that probiotics produced by *Staphylococcus epidermidis* downregulate ultraviolet light-induced IL-6 *via* a short-chain fatty acid receptor (Keshari et al., 2019).

Thus, the results presented here argue that a strong link exists between the immune system, the skin microenvironment, and the population of microorganisms selected by this environment. To our knowledge, this report is the first to demonstrate this link between IL-6 expression and the skin microbiome, revealing large-scale phylogenetic shifts following loss of this cytokine and arguing that the loss of this cytokine significantly alters the microenvironment of the mouse skin.

## Conclusion

The skin is a useful model for innate immunological and microbial interaction as it is the initial defense mechanism and protective interface from invading organisms, toxins, and physical stress. Although the bacteria–host interactions have been studied in depth, much remains unknown regarding what role genetic predisposition and IL-6 may play in influencing the cutaneous immune transcriptional profiles the microbial communities present on uninjured skin. As IL-6 represents a keystone cytokine in infection, cancer, and inflammation and can potentially drive disease progression, it is essential to establish how IL-6 contributes to maintaining cutaneous integrity as well. This study highlighted the cutaneous transcriptional diversity between commonly used laboratory mouse strains and also that the deletion of one highly pleiotropic cytokine produces a completely different repertoire. For instance, IL-6 deficiency caused the elevated expression of gene signatures such as *S100a*, *Crnn*, *Alox8*, *Acod*, *CXCL2*, *CXCR2*, *IL-1ß*, and *Trem1*, which together may correlate with increased inflammation to combat the increased bacterial load. As this model provides opportunities to perturb, engineer, and study host–microbiome interplay with a level of experimental control that is not typically achievable in human studies, there are some caveats that arise. Factors that govern skin microbial community composition among mammals are poorly understood; however, it has been shown that human skin is distinct, not only from other primates but also from other mammalian orders, further highlighting the difficult task of correlating murine to human studies (Ross et al., 2018). Although many of these current experiments were performed in murine models, they are still relevant to humans, as many of the pathways underlying inflammation and immunity in murine skin appear relevant in human infection and disease. These IL-6-deficient data could potentially correlate with human studies and the activity of IL-6-associated single nucleotide polymorphisms. As such, the most frequently studied polymorphism, SNP −174C>G, is prevalent in 40% of the general population, associated with varied transcriptional rates of IL-6, and linked to various diseases like Alzheimer’s disease, atherosclerosis, cardiovascular disease, carcinomas, insulin-independent diabetes mellitus, osteoporosis, sepsis, and systemic complications of juvenile chronic arthritis (Fishman et al., 1998; Rauramaa et al., 2000; DeMichele et al., 2003; Jeremic et al., 2014). While additional functional studies such as histology, barrier function, mechanistic testing, and microbial pathogenicity studies are necessary for further interpretation of our results, it is well beyond the scope of this manuscript. This initial investigation explores the factors that mediate this host–microbial relationship in normal, homeostatic skin and is a novel and compelling line of research that could potentially provide further understanding of how commensal bacteria become pathogenic and/or contribute to the numerous inflammatory diseases that affect the skin as well as an individual’s susceptibility.

## Data Availability Statement

The datasets presented in this study can be found via accession number PRJNA692120 on the SRA/BioProject database.

## Ethics Statement

The animal study was reviewed and approved by OUHSC IACUC.

## Author Contributions

LL-C designed the study, performed all animal work, analyzed all the data (in all treatment groups), and wrote the manuscript. CK performed the bacterial genomic experiment and analysis. JG performed library preparation and sequencing and assisted with data analysis. AG, WM, and RG contributed to critically revising and providing final approval of the manuscript. All authors contributed to the article and approved the submitted version.

## Conflict of Interest

The authors declare that the research was conducted in the absence of any commercial or financial relationships that could be construed as a potential conflict of interest.

## Footnotes

1 http://david.abcc.ncifcrf.gov/

2 www.pantherdb.org

## Supplementary Material

Supplementary Figure 1 Functional overview. **(A,B)** Gene ontology terms and **(C,D)** canonical pathways associated with genes differentially modulated in: **(A,C)** BALB/c versus C57BL/6 normal skin and **(B,D)** IL-6KO compared to C57 normal skin.

Supplementary Figure 2 16S rRNA sequencing was performed on BALB/c, C57BL/6, and IL-6 deficient (KO) skin samples and relative abundance of bacterial present on the skin is presented (*n* = 6/experimental group). Taxonomy presented at the genus level.

Supplementary Figure 3 LEfSe analysis of the microbial communities between the three microbial communities (BALB/c, C57, and IL-6 KO) show with complete known taxonomy (*p* < 0.05).

Supplementary Table 1 LEfSe analysis results of the microbial skin populations associated with BALB/c, C57, and IL-6 KO mice (Segata et al., 2011).
