# Supplementary figures and images for: Loss of Interleukin-6 Influences Transcriptional Immune Signatures and Alters Bacterial Colonization in the Skin
Source: Front Microbiol. 2021 Jul 6;12:658980. doi: 10.3389/fmicb.2021.658980 (PMC8290525; doi:10.3389/fmicb.2021.658980)

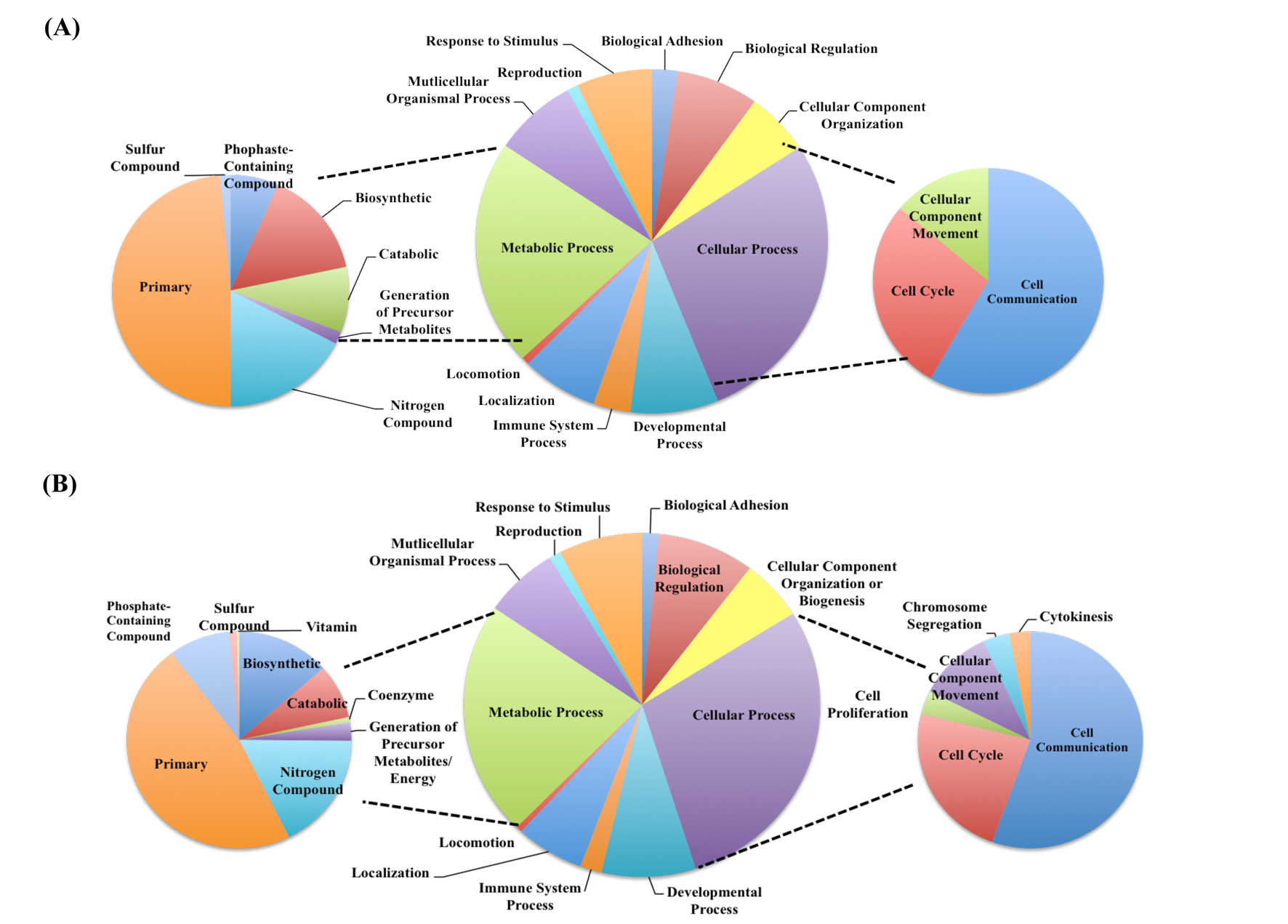

Supplement: Supplementary file 1 [file Image_1.TIF]

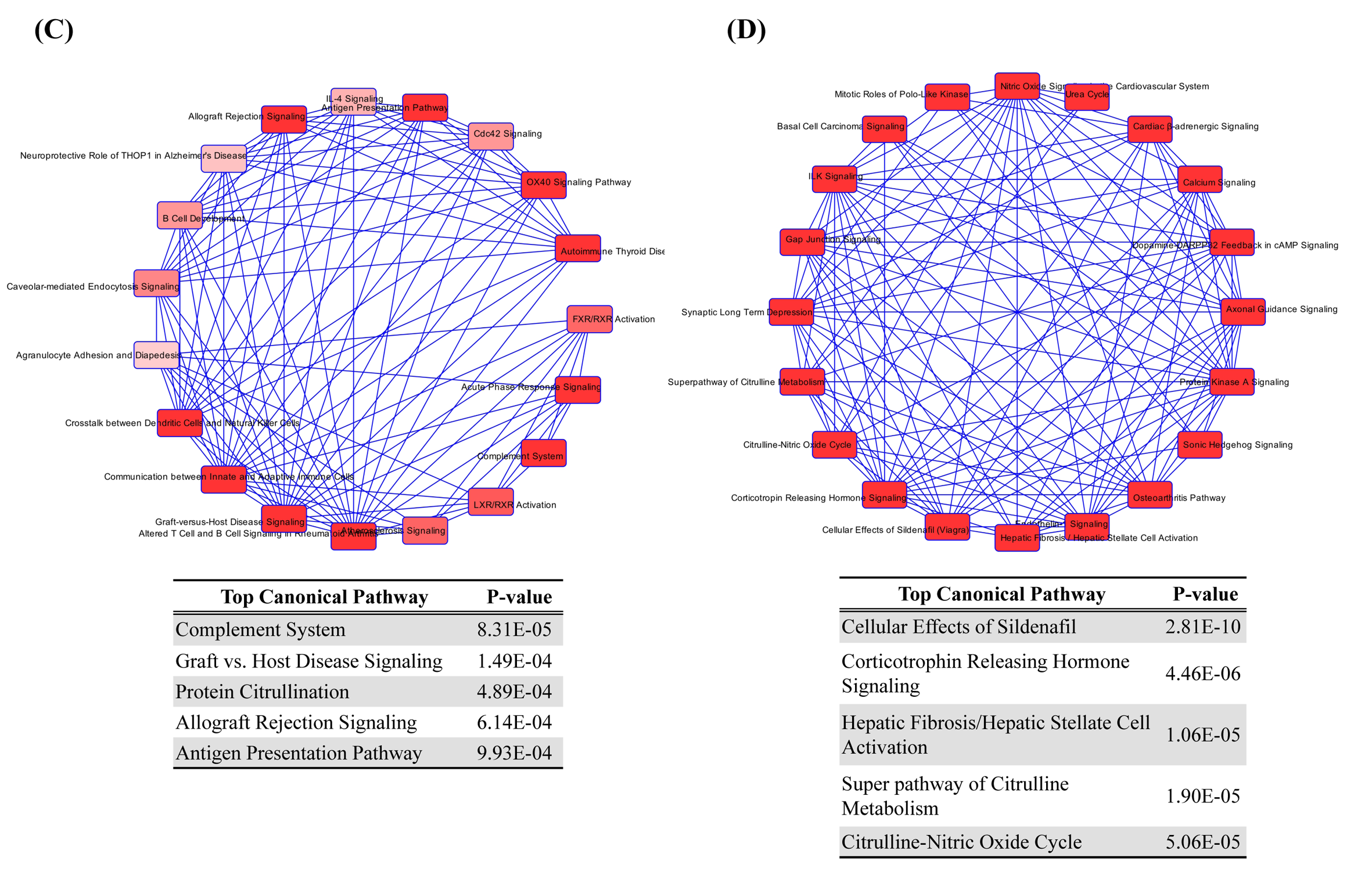

Supplement: Supplementary Figure 1 — Functional overview. (A,B) Gene ontology terms and (C,D) canonical pathways associated with genes differentially modulated in: (A,C) BALB/c versus C57BL/6 normal skin and (B,D) IL-6KO compared to C57 normal skin. [file Image_2.TIF]

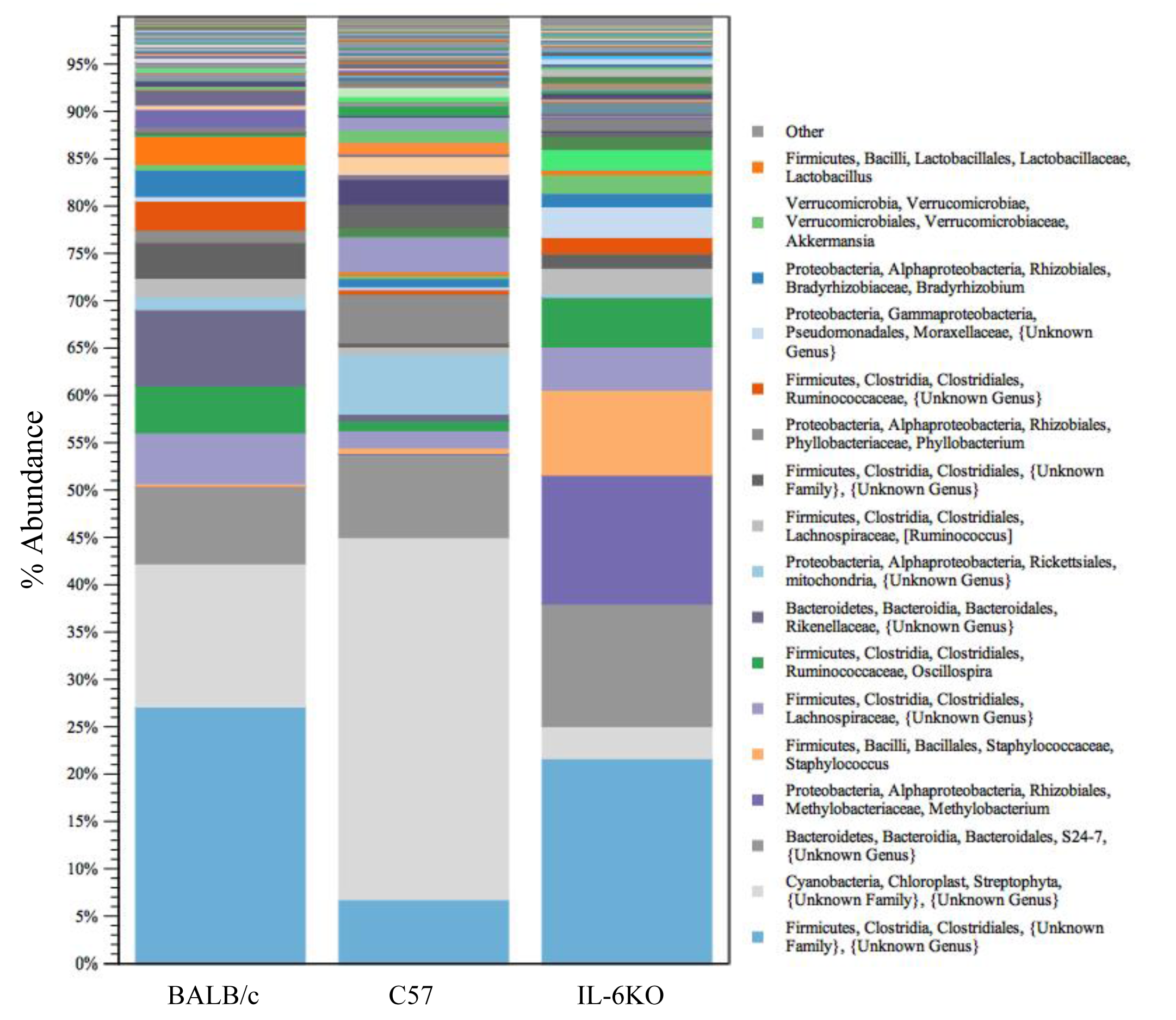

Supplement: Supplementary Figure 2 — 16S rRNA sequencing was performed on BALB/c, C57BL/6, and IL-6 deficient (KO) skin samples and relative abundance of bacterial present on the skin is presented (n = 6/experimental group). Taxonomy presented at the genus level. [file Image_3.TIF]

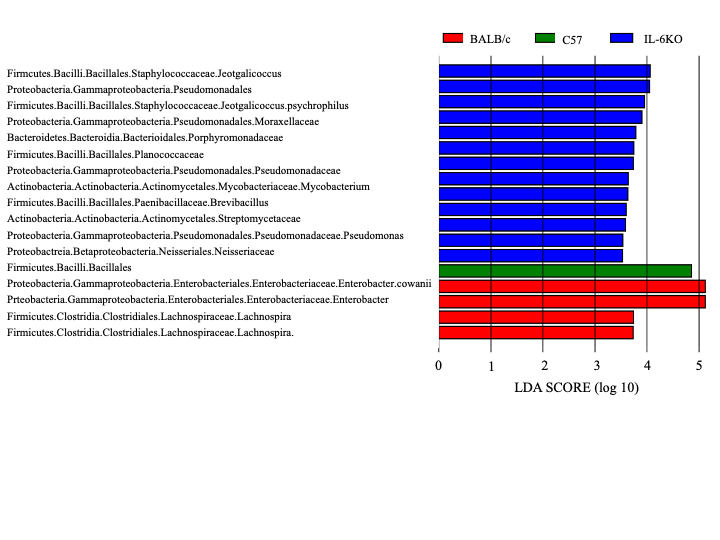

Supplement: Supplementary Figure 3 — LEfSe analysis of the microbial communities between the three microbial communities (BALB/c, C57, and IL-6 KO) show with complete known taxonomy (p < 0.05). [file Image_4.TIFF]
